# On the middle ground between open source and commercial software - the case of the Newbler program

**DOI:** 10.1186/gb4173

**Published:** 2014-04-29

**Authors:** Alexander J Nederbragt

**Affiliations:** 1 Department of Biosciences, Norwegian High-Throughput Sequencing Centre (NSC), Centre for Ecological and Evolutionary Synthesis (CEES), University of Oslo, Oslo, Norway

## 

Earlier this year, I started a petition (http://flxlexblog.wordpress.com/2014/01/31/make-newbler-open-source/) to ask Roche Applied Science to make the source code of their Newbler software (GS De Novo Assembler, GS Reference Mapper and GS Amplicon Variant Analyzer) open source. In this column, I want to explain my motivation, describe my interactions with Roche, and place this petition in the wider perspective of closed- versus open-source software.

First, I am a software user, not a developer. I do write code, but more in the form of scripts to tie existing pieces of software together, or to analyze and compare results from different programs. I have a policy of using open-source software for my scientific work. I am a firm believer in the importance of reproducibility in science, and the openness and transparency of open-source software are crucial for this. ’Non-availability of code [is] a serious impediment to reproducibility’ [1].

One of the exceptions to my rule of only using open-source software in my research is my use of the Newbler program. This software is developed and maintained by 454 Life Science, a Roche company. Newbler’s purpose is the analysis of data coming from the 454 GS FLX and GS Junior sequencing machines sold by 454 through Roche. Newbler is closed source. However, it is not a strictly commercial program as it is distributed without cost to anyone who asks (by submitting a form through http://454.com/contact-us/software-request.asp). It is a free program, but ‘free’ as in ‘for no cost’ (like ‘free beer’), not as in 'free speech' (see https://www.gnu.org/philosophy/open-source-misses-the-point.html).

In my experience, Newbler is one of the best programs for working with 454 data and it has been used for many large and small genome assemblies. For example, we used it successfully for assembling the first genome assembly of Atlantic cod [2]. I am such a fan of the program that I even wrote a user-focused manual for it (available at https://contig.wordpress.com).

I feel the fact that Newbler is not open source has hampered its use among scientists. Although I have no direct evidence for this statement, it is a fact that a number of studies that have compared programs for the assembly of sequencing data, such as GAGE (http://gage.cbcb.umd.edu/assemblers/index.html) and GAGE-B (http://ccb.jhu.edu/gage_b/genomeAssemblers/index.html) did not include the Newbler program. Nevertheless, Newbler scored very well in a competition in which outsiders could submit assemblies (assemblathon2 [http://www.gigasciencejournal.com/content/2/1/10]). Newbler is also one of the few programs that can handle data from multiple technologies for so-called hybrid assembly. Newbler can even assemble human genomes, as the company demonstrated in 2013 (http://www.roche.com/media/media_releases/med_dia_2013-02-22.htm).

I started the petition to ask Roche to make Newbler open source for the following reasons:

1) Only open-source software allows for transparent reproducibility in science, so a widely used program such as Newbler should be open source

2) In October 2013, Roche announced it will shutdown its 454 sequencing business in mid-2016 (http://www.genomeweb.com/sequencing/roche-shutting-down-454-sequencing-business); this announcement led me to worry that Newbler could disappear too

3) The value of Newbler would be much greater if researchers could learn how the program works, and perhaps even improve upon it.

In just two weeks, 162 people signed the petition (see http://figshare.com/articles/Petition_make_Newbler_open_source/936937), many more than I anticipated. In the mean time, I had contacted Roche and they agreed to let me hand over the petition during the Advances in Genome Biology and Technology (AGBT) meeting in Florida on February 13th this year. I had an open and constructive dialogue with the head of the Roche Sequencing unit and the vice president of the Roche 454 sequencing business. Roche clearly appreciated the petition initiative, and the strong signal that was sent through it. I was promised an official response, which I subsequently received and posted on my blog (http://flxlexblog.wordpress.com/2014/03/17/make-newbler-open-source-the-roche-response-and-the-future-of-newbler/). The response boils down to this: the Newbler software will be available after the 454 shutdown, free of charge as before, but Roche will not make the code open source. Roche intends to integrate it into their future sequencing platforms (they surely are working on something, alone or with a partner).

My interaction with Roche was very positive and, in a way, their response was reassuring: Newbler will not disappear. Unfortunately, we did not achieve what the signees and I hoped for: Newbler will remain closed source. This was of course a disappointment. In all fairness, however, Roche’s position is understandable. They have invested, and continue to invest, in this software, which gives their platform a competitive advantage. Releasing the code will give at least one of their competitors (IonTorrent/Thermo Fisher Scientific) an advantage as it sells instruments - the Ion Torrent PGM and Proton - that produce a similar type of data.

It is interesting to note that Newbler is positioned in the middle between commercial, closed-access and open-source software. There is no fee for using it, which makes it very accessible and provides it with a clear advantage over commercial software. Nevertheless, Newbler suffers from many of the disadvantages of closed-source software: these are black boxes, with little to no insight available into the algorithms behind them or their inner workings. There are no peer-reviewed papers describing them. Old versions are often not available, hampering reproducibility. If the company decides to pull the software off the market, or to remove a feature that is important for some researchers’ work, there is nothing that can be done. The closed-source, no-fee model has another potential problem, as there may not be an incentive for the company to provide support for the software. Luckily, this seems not to be the case for Newbler; Roche says that they record all bug reports and feature requests, and take these very seriously during development of the next version. There is, however, no transparency: their bug and feature trackers are as closed as the source code.

Interestingly, other companies who sell sequencing instruments have made important parts of the software they produce open source. Worth mentioning are Pacific Biosciences’ SMRT Analysis package (http://www.pacbiodevnet.com and https://github.com/PacificBiosciences), Thermo Scientific’s Ion Torrent mapping and variant calling software (https://github.com/iontorrent) and Illumina’s ISAAC aligner (https://github.com/sequencing). These companies clearly believe in the benefits of open-source software as part of their business model.

There will always be a market for commercial software, because of its ease of use and sometimes because it is just plainly better. I am a strong believer in open-access software, and I believe this model has a bright future. Good, closed-source but free programs such as Newbler, which are in the middle of these two extremes, will surely have a user base. But Roche have missed a huge opportunity by not making Newbler a true open-source software. Not charging for closed-access software does not necessarily make it better than commercial software.

By the way, the petition to convince Roche to make Newbler open source is still open, feel free to add your signature! (https://docs.google.com/spreadsheet/viewform?formkey=dHRvZDFUcldvZXVnWmhvSnlMWDBLQ1E6MA).
